# Analysis of the Security and Reliability of Cryptocurrency Systems Using Knowledge Discovery and Machine Learning Methods

**DOI:** 10.3390/s22239083

**Published:** 2022-11-23

**Authors:** Zeinab Shahbazi, Yung-Cheol Byun

**Affiliations:** 1 Department of Mathematics & Informatics, University of Barcelona, 08007 Barcelona, Spain; 2 Department of Computer Engineering, Major of Electronic Engineering, Institute of Information Science & Technology, Jeju National University, Jeju 63243, Repblic of Korea

**Keywords:** blockchain, knowledge discovery, machine learning, artificial intelligence, cryptocurrency

## Abstract

Cryptocurrency, often known as virtual or digital currency, is a safe platform and a key component of the blockchain that has recently attracted much interest. Utilizing blockchain technology, bitcoin transactions are recorded in blocks that provide detailed information on all financial transactions. Artificial intelligence (AI) has significant applicability in several industries because of the abundance and processing capacity of large data. One of the main issues is the absence of explanations for AI algorithms in the current decision-making standards. For instance, there is no deep-learning-based reasoning or control for the system’s input or output processes. More particularly, the bias for adversarial attacks on the process interface and learning characterizes existing AI systems. This study suggests an AI-based trustworthy architecture that uses decentralized blockchain characteristics such as smart contracts and trust oracles. The decentralized consensuses of AI predictors are also decided by this system using AI, enabling secure cryptocurrency transactions, and utilizing the blockchain technology and transactional network analysis. By utilizing AI for a thorough examination of a network, this system’s primary objective is to improve the performance of the bitcoin network in terms of transactions and security. In comparison to other state-of-the-art systems, the results demonstrate that the proposed system can achieve very accurate output.

## 1. Introduction

Blockchain and AI integration has emerged as one of the disruptive technologies that directly influence human lives. One of the key forces behind global innovation is these technologies. In recent years, the globe has had to deal with a brand-new tradable system called cryptocurrencies. For the purposes of conducting transactions through this network, this system is built on a distributed, decentralized blockchain network. In relation to the rapid advancement of blockchain technology, cryptocurrencies have also acquired notoriety and interest. Nearly 7000 different types of cryptocurrencies were traded frequently in the second quarter of 2020, with a market valuation of over USD 300 billion. Artificial-intelligence technology has a versatile impact on a variety of sectors and industries. The intricate design of AI demonstrates the findings of more than 70 years of research and study in an area that has numerous ethical and security concerns [1,2,3,4]. Adversarial attacks and biases are the two fundamental drawbacks of AI systems, according to [5,6,7,8]. The sensitivity of biases increases in the subset of data in terms of accurate decision making from AI systems, but it does not work well across the entire population. Moreover, one of the largest problems in recent years, particularly in fields of important missions, has been the lack of data explanation in AI systems. For instance, when it comes to chatbots and object identification, proposals from deep learning systems are accepted without any assumptions. Games with a chance of a wrong guess are not particularly stringent. The situation is urgent, directly affecting substantial assets or human lives, and it is still a concern when it comes to incorrect prediction and decision making in healthcare systems, security, or finance.

**Proof of Work (PoW):** PoW is one of the blockchain’s consensus methods for adding new transactions to the ledger. Verifying transactions and adding new blocks to the chain are the first objectives. PoW initiates the process of participants completing transactions into the network and receiving rewards. Individuals are identified as miners, and mining as the activity. Digital tokens are sent between miners over the network, and in a decentralized network, all transactions are collected in a block. The purpose of this entire procedure is to arrange the blocks, verify the transactions, and reach minor consensus.

**Proof of Stake (PoS):** The consensus protocol for addressing the issue of energy usage is called PoS. A blockchain network’s stakeholders can produce new blocks thanks to this protocol. The protocol chooses the validators on the basis of different criteria such as delegated validators and high-frequency transacting validators. In addition to being more energy-efficient than PoW, PoS also addresses some security issues by avoiding the allocation of validators who own blockchain-native money. The generation of new blocks is delayed if validation fails. When it comes to detecting changes and data streaming, PoS offers the possibility of delay tolerance in AI applications.

The revolution of the digital currency is mentioned in [9,10,11]. Different central bank digital currencies (CBDC) variations were taken into consideration to prsent this system’s decision making, dangers, and benefits. A review of the bank-backed digital money is given in [12]. This report analyzed current CBDC disputes and focused on the nations that are opposed to CBDC implementation. The authors in [13] evaluated a novel situation involving the strength of mining in Bitcoin and Ethereum. There are a total of six scenarios offered for the parameters of the network data collected from mining hardware efficiency. The results indicate that the overall power of blockchain demand is constrained by the mining equipment’s efficiency. The energy qualification of cryptocurrency mining was mentioned in [14,15]. In this method, minimal power evaluation needs in terms of energy consumption for producing the value element of digital assets were evaluated for Bitcoin, Ethereum, Litecoin, and Monero. In [16], the authors primarily focused on bitcoin mining and the environment in sustainable nations. The environmental performance index (EPI), the price of energy, the temperature, human capital, and legislative restrictions are all taken into account at the beginning of the process. The most recent study about cryptocurrencies and the blockchain is the one in [17,18]. The history, definition, and elements that affect the value, legal status, and other aspects of cryptocurrencies were all discussed.

The following is a summary of this paper’s significant contributions:

- The network modeling classification of semantic edges and nodes, and the network creation of transactional information for various purposes.
- Presenting a network summary based on network evaluation, properties, and market effects.
- Presenting an overview of AI in the context of cryptocurrencies and a thorough examination of the transactional network.
- The techniques used in blockchain and AI approaches include entity recognition, activity detection, and transactional tracing, delivering reliable records for the performance assessment of the network.

The remainder of this paper is organized as follows: Section 2 provides a full summary of the literature review pertaining to the framework of AI and cryptocurrencies. The mechanism for knowledge discovery in this framework, which is based on system transactions, is presented in Section 3. The outcomes and environmental data of the suggested approach are presented in Section 4, and we conclude this paper in Section 5.

## 2. Literature Review

The state of the art in knowledge discovery and AI through the blockchain architecture is briefly described in this section. There are two distinct sections: Blockchain for AI, and cryptocurrency-based exchange security.

### 2.1. Blockchain for AI

According to [19,20], the blockchain framework is a well-liked safe framework for transactions between users and many different businesses. Blockchain technology is a decentralized network that empowers the market with many AI components, including data, processing power, and algorithms. Decentralized AI is a novel idea for the blockchain and AI combination, as noted in [19,21,22,23]. This method raises the bar for AI innovation and adaptation. Additionally, the blockchain impacts AI transparency, dependability, and judgments because of the publicly accessible dataset, which boosts the framework’s confidence and privacy [24,25,26,27,28]. Secure data sharing, which is an AI revolution in terms of vast data and the gold standard in the economics of data-driven services, is another benefit of the blockchain. In terms of decision making, smart-contract-based systems produce an exact and trustworthy system that validates and verifies blockchain nodes. This kind of choice cannot be thrown down or followed and traced by the participants of the entity. Among well-known decentralized storage technologies are Filecoin [29], Storj [30], Interplanetary File System [31], and BigChainDB [32]. The blockchain for AI issues was reviewed in [33,34]. Developing platform and AI-targeted blockchain applications were the key focal points. The difficulties and implications of the blockchain on AI have been discovered and explored. Decentralized AI, which enables trust and decision making, processes and performs analyses, and secures shared information that is recorded in blockchain is the integration of AI and blockchain [35,36]. The difficulties, solutions, and future directions of the administration of the energy cloud based on the blockchain and AI were given in [37]. The system demonstrates how the blockchain and AI may work together to resolve privacy and security issues by utilizing the decentralized AI energy cloud management (ECM) architecture.

### 2.2. Cryptocurrency-Based Exchange Security

On the basis of the sender’s private key that determines the type of money, transactions using cryptocurrencies transmit a pervasive message to the blockchain network, which is addressed in [38]. No one is able to alter the data once the private key signs a message, acknowledging the transaction to the blockchain network and nodes. All data are deactivated if the private key is stolen. Problem-solving and encryption algorithms are the current study fields that have received the most attention [39,40,41,42,43]. Cryptocurrency adoption has increased dramatically over time and is growing more popular among the younger population. It is referred to as the currency of the new digital age. In [44], the authors examined the prevalent cryptocurrency systems, and the disruptive innovations and technologies that underpin them. For the purpose of predicting cryptocurrency prices, the authors in [45] proposed the reliable DL-Gues framework, which takes into account the interdependence of each coin, and the market sentiment. Using the price history and tweets of Dash, Litecoin, and Bitcoin, and numerous loss functions, we took into consideration Dash price prediction. Additionally, using the price history and tweets of Bitcoin-Cash, Litecoin, and Bitcoin, we inferred the findings for price prediction in order to test the applicability of DL-GuesS on additional cryptocurrencies. Fault tree analysis was the method used in [46] to describe dependability and analyze the architecture of blockchain oracle systems. Weak links that affected the overall reliability of a blockchain-based system could be found by calculating the reliability of oracle methods.

One of the most important recent research papers [47,48,49] focused on the statistical perspective problem. The suggested method lowered the threshold of the control mechanism in terms of the hot wallet to lessen the refilling records for the cold wallet and prevent the representation of private keys in the cold wallet during transfer. The most recent blockchain-based artificial-intelligence (AI) applications are shown in Table 1. In terms of goals, advantages, and applied scenarios, the table contrasts augmented and lean data learning, hybrid models, digital twins, explainable AI, and automated machine learning. The application of blockchain and AI systems together with the trust verification of the utilized dataset is the major goal of the systems. The key idea of the Bitcoin blockchain’s feature was introduced in [50]. The highlights demonstrate the blockchain’s use in banking and finance in terms of this framework’s IoT adaptation.

## 3. Research Methodology

**Figure 1:**
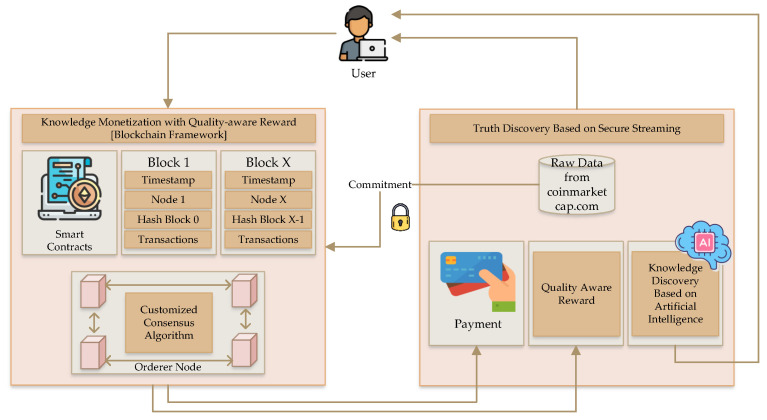
Overview of the proposed knowledge discovery in a blockchain framework.

The primary goal of this research is knowledge discovery on blockchain-based and AI-based cryptocurrency systems to improve the security and dependability of any exchanges over this network. The knowledge discovery method reveals information and architecture that are hidden from the user’s view. The proposed system’s intricate architecture, which employs a combined method in cryptocurrencies, is shown in Figure 1.

An overview of knowledge discovery utilizing AI within the blockchain framework is provided by the designed architecture. This commitment, which offers a secure knowledge discovery process, involves two key elements. The blockchain framework includes a customized consensus algorithm linked to blocks with ordered nodes and smart contracts to maintain the system’s transactional records.

### 3.1. Data

The comprehensive information and records related to the blockchain dataset are available online for unheard network transactional analysis. In this process, we collected the dataset from the https://coinmarketcap.com/ website, which contains all information related to the cryptocurrencies, market caps, volumes, etc. Table 2 presents information related to the collected dataset for the trustworthy processing of knowledge discovery and framework based on the four cryptocurrency types of Bitcoin, Ethereum, Litecoin, and Monero.

### 3.2. Data Transactions Handled in Real-Time Dynamic Environments

**Figure 2:**
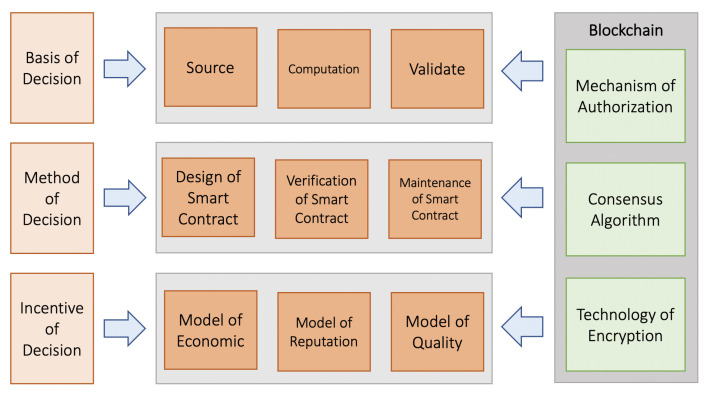
Cryptocurrency management framework.

The decentralized autonomous organization (DAO) is a model organizational structure built on blockchain technology that stands out for its openness and independence. The DAO automates and independently creates management decisions by using coded and programmed smart contracts to break down complex management functions into a variety of computer words. Crypto management adopts distributed ownership using DAO as the organizational form and management structure, making all members the organization’s owners and decision makers. In DAO, crypto management is heavily reliant on distributed collaboration and collective intelligence, which can circumvent the limitations of hierarchical management structures and individual-dependent decision-making models, thus improving intelligence and dependability. The independence of crypto administration is built on concepts that can be effectively created and dynamically performed. They typically outline the complicated interactions between people and organizations, the method for managing and allocating resources, and the objectives and limitations of stakeholders. DAO uses multiparty negotiation, games, and even votes to obtain the ultimate consensus. The terminology used to describe various organizational management functions may be translated into contract rules using a set of standardized standards, and many interactive rules can be combined to fully specify complicated management rules. As a result, under the established DAO principles, crypto administration can work freely and efficiently. To give organizational management a different collective decision framework and system of governance, crypto management leverages the DAO. The DAO’s governance framework must be created to meet the objectives of fairness, efficiency, collaboration, security, cost-effectiveness, and other aspects of crypto administration. The two key components are collaboration mode, which directs any nodes to participate actively in proposals, and decision mode, which controls all nodes to reach the agreed-upon outcome. Particularly when a conflict emerges, an efficient dispute resolution process is required to prevent self-serving suggestions and criminal collaboration, protecting both individual interests and communal security. Individual intelligence blockages at every stage impact the outcomes in traditional top–down and bottom–up hierarchical organizational decision-making frameworks, causing some innovative and advantageous decisions to be mistakenly abandoned. This also impairs the organization’s management effectiveness and capacity for growth. Through the blockchain-based DAO, crypto management enables multiparty nodes to collaborate on voting and proposition processes for decisions. It is by definition a democratic form of governance where the entire community participates. Throughout this procedure, it is important to select a voting mechanism that is acceptable for the governance framework in place. This management framework outlines how to gain voting qualifications and how to use them to voice opinions on organizational management. Figure 2 presents crypto data management decision effects and efficiency.

### 3.3. Knowledge Discovery Using Artificial Intelligence

Regarding the latest deep-learning (DL) models, classification and detection processes can achieve inimitable performance among ML models. DL models are difficult to certify, debug, and interpret, and they are not able to give proper descriptions for validating models. From another point of view, AI changes the system concept into symbols or rules, e.g., knowledge graphs, which are easy to present.

**Figure 3:**
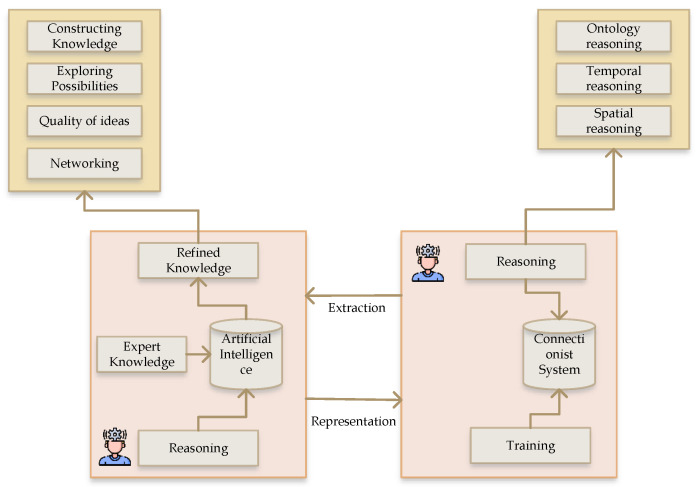
AI learning cycle framework.

Figure 3 gives an overview of the proposed system’s AI framework. The connection between AI and the connectionist system is based on extraction and representation, which refine knowledge from the system and similarly perform the reasoning process. The reasoning process follows constructing knowledge, exploring possibilities and the quality of ideas, and networking.

### 3.4. Knowledge Discovery Using Blockchains

The security and trustworthiness of the information are two of the main challenges of the users during this decade due to much illegal online processing. In this system, the main focus is to discover trustworthy knowledge from a blockchain framework. The blockchain is one of the secure platforms that contain various consensus protocols and different architectures such as multiledgers, simple ledgers, and interprobability.

**Figure 4:**
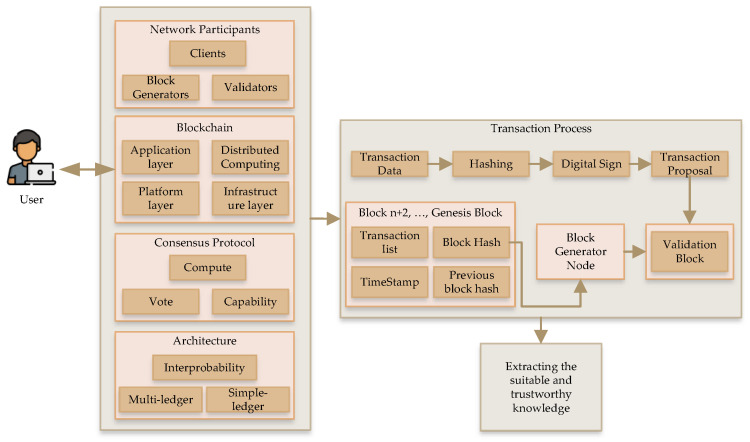
Knowledge discovery based on a blockchain framework.

**Figure 5:**
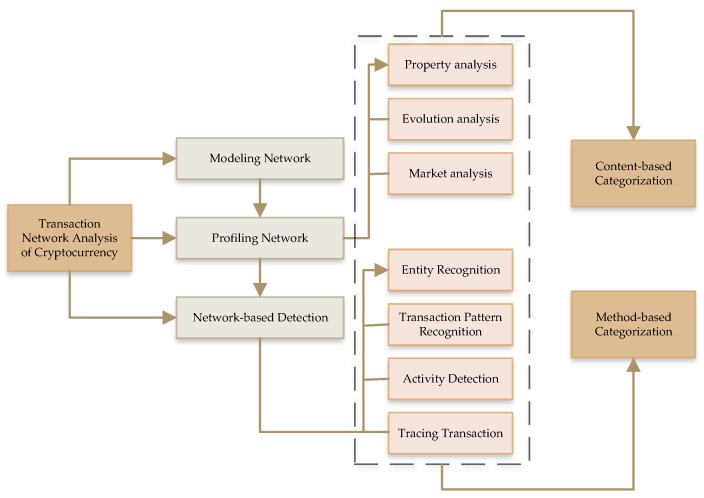
Network analysis of cryptocurrency transactions.

Figure 4 shows the knowledge discovery process from a blockchain framework. There are four layers, namely, network participants, blockchain framework process, consensus protocol, and architecture, which process a user request before a transaction in the system. Figure 5 shows the transactional network analysis of cryptocurrency. There are three categories: modeling the network, profiling the network, and detection based on network. In this procedure, there are three types of analysis: property, evolution, and market analyses. Similarly, there are two types of recognition processes: entity recognition and transactional-pattern recognition. Lastly, there are activity detection and tracing transaction.

### 3.5. Security Analysis of Data Integrity Transactions in Blockchain Network

The suggested framework was utilized to ascertain and establish what occurred in cases where there was disagreement regarding who was responsible for an incident. The integrity of the data that are accessible must be guaranteed by the company handling a claim or a law enforcement official responding to an incident. Once data integrity is confirmed, it is possible to conclusively identify the responsible party. Using our architecture as a framework, the investigator or agent begins by gaining access to the relevant forensic data that are kept in the data center. Then, they must compile the supplied transactions that include the data hashed for the first-level blockchain systems, along with their associated hashed root values and hashed routes. The investigator or agent could confirm that the data center provided authorities with valid/tamperproof IoT hash data if the quantity is indeed saved in the blockchain and the computed hashed root match. Additionally, numerous multichain miners have independently verified the existence of the transaction on the blockchain, and that significant PoW/computational time has been set aside to guarantee the integrity of hash data in a multichain system.

### 3.6. Taxonomy

This section discusses key blockchain concepts in AI applications. There are five key concepts in blockchain AI integrated systems: consensus protocols, decentralized infrastructures, decentralized AI operations, types, and decentralized AI applications.

#### 3.6.1. Knowledge Management and Discovery

One of the decentralized AI applications is the knowledge management and discovery process, which is very famous in modern AI applications for managing a huge number of data. This process offers the benefits of systemwide and application-wide intelligence for customizing knowledge patterns in special applications, users, and devices. The decentralization of knowledge management and discovery provides knowledge patterns based on personalized information regarding the stakeholders’ needs. A blockchain framework provides a secure knowledge-transfer tracing process between an AI system and application stakeholders.

#### 3.6.2. Learning and Reasoning

The learning process is the most important part of knowledge discovery, and it solves the various machine-learning problems such as regression, classification, clustering, and pattern mining. Moreover, the blockchain provides unalterable high-security learning models based on historical data. Another aspect of AI is logic programming, which is very important for developing the rules for reasoning and generalizing the application’s components. During this process, the blockchain framework comes with distributed reasoning for facilitating a personal reasoning strategy. Similarly, smart contracts ensure the availability of unforgettable reasoning for future processes in this strategy.

## 4. Experimental Results and Development Environment

This section provides the development environment of the proposed knowledge discovery in the blockchain framework. The main concept of this research is the integration of AI into the blockchain to overcome the trustworthiness issue in cryptocurrency. Table 3 shows the information of the applied process and components in this system. The operating system was Microsoft Windows 10, CPU is Intel(R) Core(TM) i7-8700@3.20 GHz. The main memory used for processing was 16 GB, the core programming language was Python, the IDE was PyCharm Professional 2020, and the machine-learning algorithm was AI.

Table 4 shows the overview of the notations used in this process.

### 4.1. Data Management

To have enough data storage, there is a need to manage the dataset on the basis of AI applications that can achieve a relevant and high level of accuracy, and the dataset must be collected from trustworthy sources. AI applications mainly focus on managing centralized data to execute the nodes from the underlying network. This process contains data filtration, segmentation, routing, and data storage in intelligent data management. Table 5 presents the integrated system feature extraction with the benefits of this combined method.

### 4.2. Performance Evaluation of Knowledge Discovery in Blockchain

The presented system aims to extract information from the blockchain framework and focuses on the trustworthiness of this system based on AI for decentralized network decision making. Equations (1) and (2) present $T_{generation}$ and $T_{verification}$, which focus on the taken time generation and verification process. Equation (3) shows the combination of processing time, transactional time, and queue time to evaluate the delay time $D_{time}$ processing.

(1) $$T_{generation}=\frac{Total\;Number\;of\;Hashes}{Hash\;Per\;Second}$$

(2) $$T_{verification}=\frac{T_{generation}}{Number\;of\;cores}$$

(3) $$D_{time}=T_{Processing}+T_{transaction}+T_{Queue}$$

**Figure 6:**
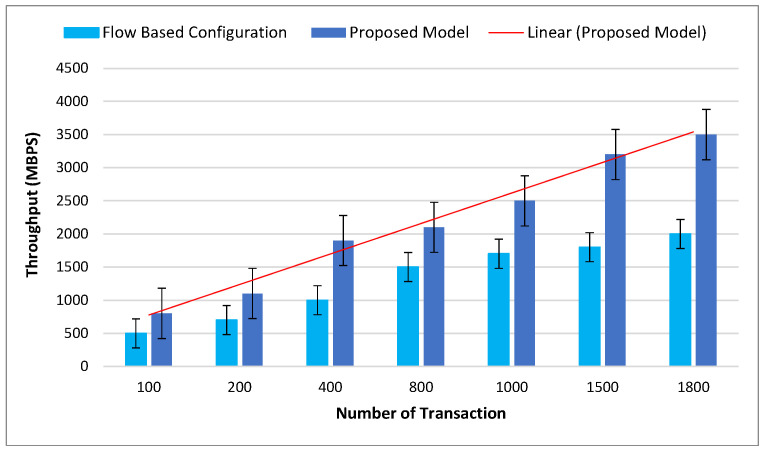
Comparison of the proposed model with throughput.

Figure 6 presents the comparison of the throughput records with the proposed model. Throughput in this process means successfully transferred data to the decided destination. The comparison is with the flow-based configuration (FBC) model. As observed from the figure, the main reason for improving the results is the structure of clustering, which reduces the overhead of the originated FBC and PoW.

**Figure 7:**
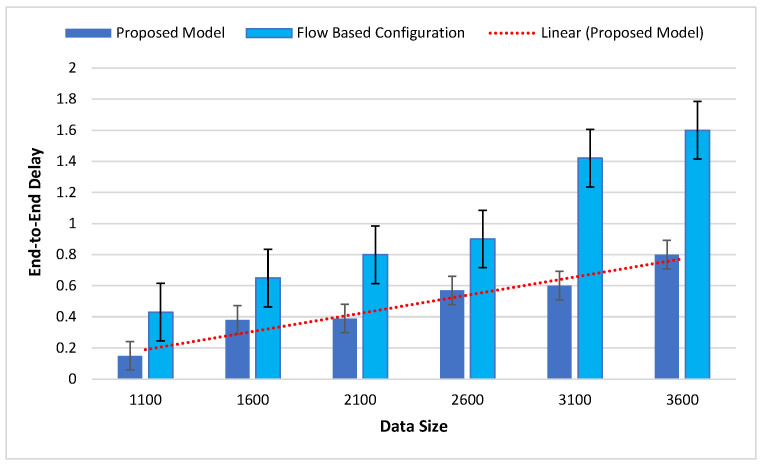
Comparison of the delay in the proposed model with flow-based configuration.

Figure 7 presents the delay rate of the FBC compared with the proposed model. Regarding the increasing number of transactions, the throughput also increases.

**Figure 8:**
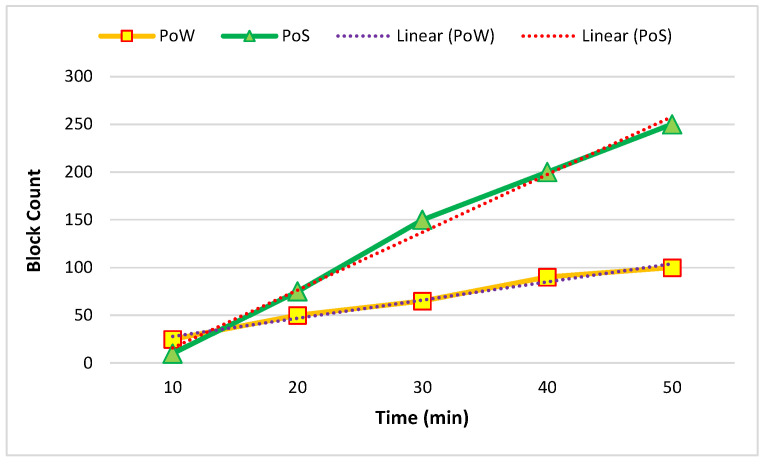
Average of five created nodes in one hour.

**Figure 9:**
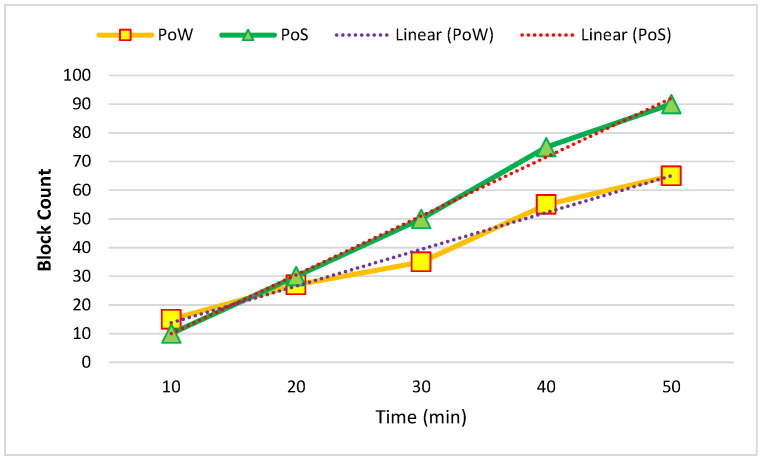
Average of ten created nodes in one hour.

Figure 8 and Figure 9 show the temporal blockchain-network performance between five and ten nodes. The required time for the generation of blocks and the block throughput were analyzed. The proof of work (PoW) and proof of stake (PoS) were based on the analyzed time in this process.

**Figure 10:**
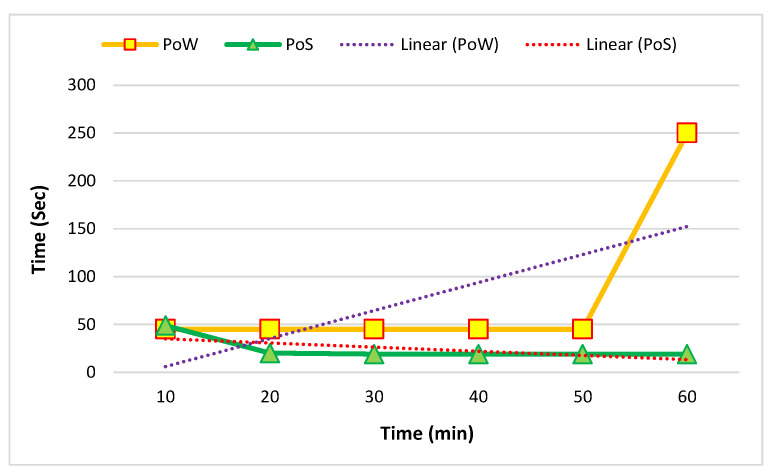
Average mining time in five nodes in one hour.

**Figure 11:**
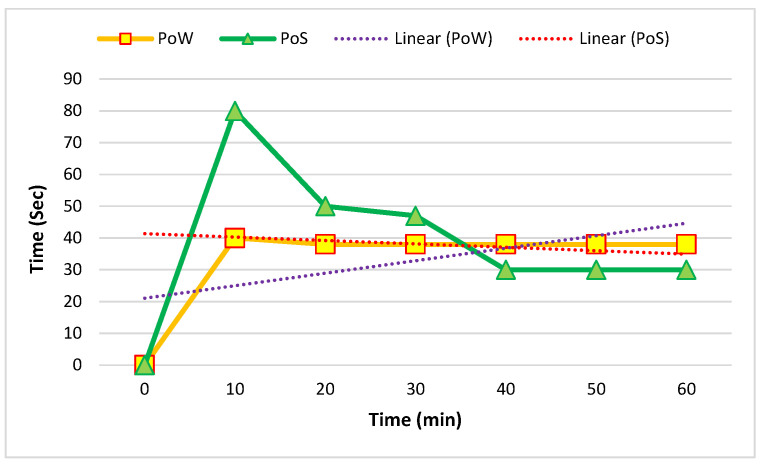
Average mining time in ten nodes in one hour.

Figure 10 and Figure 11 show the comparison of the average mining time of five and ten nodes. The mining time was based on minutes and seconds.

**Figure 12:**
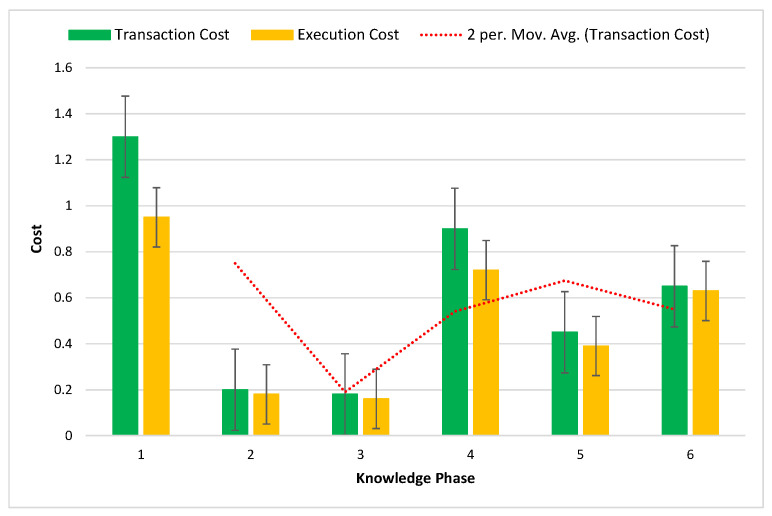
Knowledge phase of transactional and execution costs of the blockchain network.

Figure 12 shows the blockchain network’s execution cost and transactional phase in this system. The comparison was in six phases in terms of knowledge phase and cost.

### 4.3. Blockchain Operation Overhead

**Figure 13:**
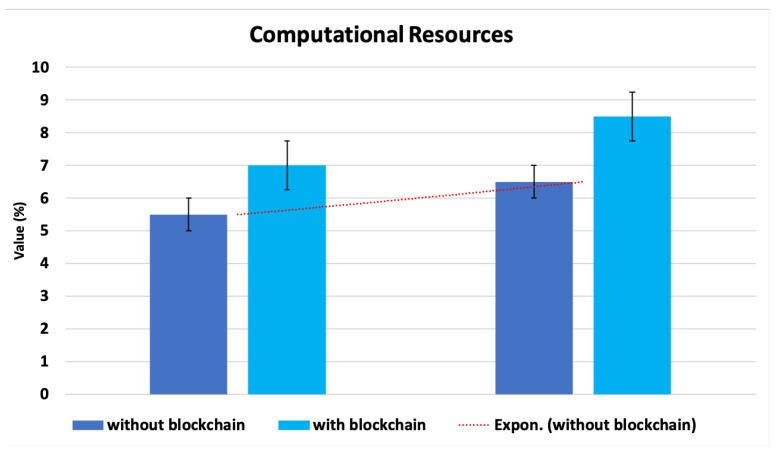
Complexity of the proposed architecture’s computation.

On the basis of the blockchain operation, we assessed the proposed architecture’s overall complexity. The blockchain process added extra overhead to the suggested architecture in comparison to centralized and distributed structures. As depicted in Figure 13, we observed the fog nodes’ typical use of processing resources (CPU and memory) during blockchain activity. In order to commit and pack the transactions into new blocks in the blockchain, the fog nodes used slightly more memory and CPU during blockchain operations. The modest increase in overhead can be tolerated because the suggested decentralized design is superior to both centralized and distributed architectures in terms of accuracy and detection time.

## 5. Conclusions

The proposed approach was designed on the basis of integrating AI and the blockchain for knowledge discovery in various cryptocurrency frameworks. The designed system focuses on reasoning, learning, and knowledge management, which directly affect the process of the system to extract the hidden part of a transactional process. The system’s effectiveness was analyzed on the basis of a real-time dynamic network to represent the system’s effectiveness. The blockchain-based system uses knowledge monetization for the confidentiality of knowledge, and automatically provides quality as a reward to the client. The results showed that the system could achieve affordable performance.Furthermore, the taxonomy and implementation comparison for AI operations with the blockchain infrastructure and protocols were discussed. According to the findings of our evaluation, the suggested decentralized security architecture performed better than the centralized and distributed architectures. Our research also indicated that the architecture could be used in conjunction with the IoT ecosystem as a security detection component that monitors and analyzes the traffic data of the entire IoT ecosystem in order to identify and prevent potential attacks.

## 6. Discussion and Future Work

The analysis of the cryptocurrency transaction network and network modeling give the ability to abstract the transnational data of a cryptocurrency regarding the objects of specific nodes and the relationship between edges and objects. There are various ways of organizing the cryptocurrencies’ transnational data with different structures, and abstracted networks change a lot. Similarly, there are different methods of modeling the network, for which a suitable model for various cryptocurrencies is adaptive low-information loss modeling, which gives identical notifications to downstream tasks. Possible future directions in this field are network modeling based on compatible transactions, information complementation based on practical networks, online learning, and network analysis based on dynamic transactions, which should be considered for further progress in this field. The blockchain technology is one of the approaches for reforming the mode of traditional industries. The growth of data is one of the aspects of this technique. Due to cryptocurrency network analysis, transactions still do not meet the requirements of practical applications, which is the main problem of the multidata source structure of the blockchain.

## Figures and Tables

**Table 1:** Latest AI application trends using blockchain technology.

Trends	Objective	Benefit	Application	Data Source
Augmented and lean of data learning [51]	Transfer learning between various AI models	Reliability, execution tracing, trust	Military, healthcare, autonomous vehicles	UDAC, D-LA dictionary
Hybrid model [52]	Combination of various ML models to get better decisions	Performance Trust Provenance	Decision-agnostic, real-time, data-source- agnostic	Real vehicle dataset
Digital twins [53]	Data translation and simulation of digital world	Reliability, trust, provenance	Offshore vessels, wind turbines, aircraft engines	Sensor data
Explainable AI [54]	Designing the interpretable and trusted transaction based on AI	Reliability Trust Execution of tracing	Military, healthcare, autonomous vehicles	Merchants’ previous sales and transactions.
Automated machine learning [55]	Using the raw data to automate the process of ML with higher speed	Immutability Permanence	Massive production, big data analysis, Industry 4.0	Featurized dataset

**Table 2:** Dataset information for the four cryptocurrency types.

Cryptocurrency	Price ($)	24 h (%)	7 d (%)	Market Cap ($)	Volume (24 h)
Bitcoin	47.197	1.11	7.59	891.255	36.184
Ethereum	3.735	1.96	8.77	443.644	12.061
Litecoin	148.27	1.77	10.57	10.271	783.729
Monero	227.93	7.60	8.59	4.115	220.810

**Table 3:** Development environment.

Module	Component	Description
Knowledge Discovery	Operating system	Microsoft Windows 10
	CPU	Intel (R) Core (TM) i7-8700@3.20 GHz
	Main memory	16 GB RAM
	Core programming language	Python
	IDE	PyCharm Professional 2020
	ML algorithm	AI
Blockchain Framework	Operating System	Ubuntu Linux 18.04 LTS
	Docker engine	Version 18.06.1-ce
	Docker composer	Version 1.13.0
	IDE	Composer playground
	Programming Language	Node.js

**Table 4:** Notations.

Notations	Description
PoW	Proof of work
FBC	Flow-based configuration
PoS	Proof of stake
AI	Artificial intelligence
ML	Machine learning
$D_{time}$	Delay time
$T_{processing}$	Processing time
$T_{transaction}$	Transactional time
$T_{queue}$	Queue time

**Table 5:** Feature extraction of the integrated system.

AI	Blockchain	Benefits of Integration
Volatile	Data integrity	Decentralized intelligence
- Knowledge - Data - Decision	Attacks Resilient	High efficiency
Centralized	Decentralized	Enhanced data security
Changing	Deterministic	Trust improvement
Probabilistic	Immutable	Collective decision making

## Data Availability

Not applicable.

## References

[1] Awad E., Dsouza S., Kim R., Schulz J., Henrich J., Shariff A., Bonnefon J.F., Rahwan I. (2018). The moral machine experiment. Nature.

[2] Frank M.R., Wang D., Cebrian M., Rahwan I. (2019). The evolution of citation graphs in artificial intelligence research. Nat. Mach. Intell..

[3] Dai W.Z., Xu Q., Yu Y., Zhou Z.H. (2019). Bridging machine learning and logical reasoning by abductive learning. Adv. Neural Inf. Process. Syst..

[4] Hitzler P., Bianchi F., Ebrahimi M., Sarker M.K. (2020). Neural-symbolic integration and the Semantic Web. Semant. Web.

[5] Chen T., Liu J., Xiang Y., Niu W., Tong E., Han Z. (2019). Adversarial attack and defense in reinforcement learning-from AI security view. Cybersecurity.

[6] Ng J. (2021). An Alternative Rationalisation of Creative AI by De-Familiarising Creativity: Towards an Intelligibility of Its Own Terms. AI for Everyone?.

[7] Tiddi I., Schlobach S. (2022). Knowledge graphs as tools for explainable machine learning: A survey. Artif. Intell..

[8] De Raedt L., Dumančić S., Manhaeve R., Marra G. (2020). From statistical relational to neuro-symbolic artificial intelligence. arXiv.

[9] Shahbazi Z., Byun Y.C. (2022). Machine Learning-Based Analysis of Cryptocurrency Market Financial Risk Management. IEEE Access.

[10] Cunha P.R., Melo P., Sebastião H. (2021). From Bitcoin to Central Bank Digital Currencies: Making Sense of the Digital Money Revolution. Future Internet.

[11] Shahbazi Z., Byun Y.C. (2022). Knowledge Discovery on Cryptocurrency Exchange Rate Prediction Using Machine Learning Pipelines. Sensors.

[12] Náñez Alonso S.L., Echarte Fernández M.Á., Sanz Bas D., Kaczmarek J. (2020). Reasons fostering or discouraging the implementation of central bank-backed digital currency: A review. Economies.

[13] Zade M., Myklebost J., Tzscheutschler P., Wagner U. (2019). Is bitcoin the only problem? a scenario model for the power demand of blockchains. Front. Energy Res..

[14] Krause M.J., Tolaymat T. (2018). Quantification of energy and carbon costs for mining cryptocurrencies. Nat. Sustain..

[15] Shahbazi Z., Byun Y.C. (2022). NLP-Based Digital Forensic Analysis for Online Social Network Based on System Security. Int. J. Environ. Res. Public Health.

[16] Náñez Alonso S.L., Jorge-Vázquez J., Echarte Fernández M.Á., Reier Forradellas R.F. (2021). Cryptocurrency mining from an economic and environmental perspective. Analysis of the most and least sustainable countries. Energies.

[17] Kar M. (2022). Blockchain Technology and Cryptocurrency: Current Situation and Future Prospects. Blockchain Technology.

[18] Shahbazi Z., Byun Y.C. (2021). Blockchain-based event detection and trust verification using natural language processing and machine learning. IEEE Access.

[19] AlShamsi M., Salloum S.A., Alshurideh M., Abdallah S. (2021). Artificial intelligence and blockchain for transparency in governance. Artificial Intelligence for Sustainable Development: Theory, Practice and Future Applications.

[20] Latif S.A., Wen F.B.X., Iwendi C., Li-li F.W., Mohsin S.M., Han Z., Band S.S. (2022). AI-empowered, blockchain and SDN integrated security architecture for IoT network of cyber physical systems. Comput. Commun..

[21] Kumaresh S. (2021). Decentralised Artificial Intelligence Enabled Blockchain Network Model. Turk. J. Comput. Math. Educ. (Turcomat).

[22] Nyame G., Qin Z., Obour Agyekum K.O.B., Sifah E.B. (2020). An ECDSA approach to access control in knowledge management systems using blockchain. Information.

[23] Schniederjans D.G., Curado C., Khalajhedayati M. (2020). Supply chain digitisation trends: An integration of knowledge management. Int. J. Prod. Econ..

[24] Hussain A.A., Al-Turjman F. (2021). Artificial intelligence and blockchain: A review. Trans. Emerg. Telecommun. Technol..

[25] Sharma Y., Balamurugan B., Snegar N., Ilavendhan A. (2021). How IoT, AI, and Blockchain Will Revolutionize Business. Blockchain, Internet of Things, and Artificial Intelligence.

[26] Lin X., Li J., Wu J., Liang H., Yang W. (2019). Making knowledge tradable in edge-AI enabled IoT: A consortium blockchain-based efficient and incentive approach. IEEE Trans. Ind. Inform..

[27] Caldarelli G., Rossignoli C., Zardini A. (2020). Overcoming the blockchain oracle problem in the traceability of non-fungible products. Sustainability.

[28] Lee K.M., Ra I. (2020). Data privacy-preserving distributed knowledge discovery based on the blockchain. Inf. Technol. Manag..

[29] Ascigil O., Reñé S., Król M., Pavlou G., Zhang L., Hasegawa T., Koizumi Y., Kita K. Towards peer-to-peer content retrieval markets: Enhancing IPFS with ICN. Proceedings of the 6th ACM Conference on Information-Centric Networking.

[30] de Figueiredo S., Madhusudan A., Reniers V., Nikova S., Preneel B. Exploring the storj network: A security analysis. Proceedings of the 36th Annual ACM Symposium on Applied Computing.

[31] Wang L., Liu X., Lin X. (2021). A Fair and Privacy-Preserving Image Trading System Based on Blockchain and Group Signature. Secur. Commun. Netw..

[32] Yang R., Yu F.R., Si P., Yang Z., Zhang Y. (2019). Integrated blockchain and edge computing systems: A survey, some research issues and challenges. IEEE Commun. Surv. Tutor..

[33] Salah K., Rehman M.H.U., Nizamuddin N., Al-Fuqaha A. (2019). Blockchain for AI: Review and open research challenges. IEEE Access.

[34] Jamil F., Ahmad S., Whangbo T.K., Muthanna A., Kim D.H. (2022). Improving blockchain performance in clinical trials using intelligent optimal transaction traffic control mechanism in smart healthcare applications. Comput. Ind. Eng..

[35] Team N.A. (2018). Decentralized Ai Blockchain Whitepaper.

[36] Dinh T.N., Thai M.T. (2018). Ai and blockchain: A disruptive integration. Computer.

[37] Kumari A., Gupta R., Tanwar S., Kumar N. (2020). Blockchain and AI amalgamation for energy cloud management: Challenges, solutions, and future directions. J. Parallel Distrib. Comput..

[38] Alkhammash I., Halboob W. (2021). A Bitcoin Wallet Security System (BWSS). ITNG 2021 18th International Conference on Information Technology-New Generations.

[39] Ziegeldorf J.H., Matzutt R., Henze M., Grossmann F., Wehrle K. (2018). Secure and anonymous decentralized Bitcoin mixing. Future Gener. Comput. Syst..

[40] Canetti R., Gennaro R., Goldfeder S., Makriyannis N., Peled U. UC non-interactive, proactive, threshold ECDSA with identifiable aborts. Proceedings of the 2020 ACM SIGSAC Conference on Computer and Communications Security.

[41] Liu X., Susilo W., Baek J. (2021). Secure Computation of Shared Secrets and Its Applications. International Conference on Information Security Applications.

[42] Liu X.F., Jiang X.J., Liu S.H., Tse C.K. (2021). Knowledge discovery in cryptocurrency transactions: A survey. IEEE Access.

[43] Biradar U.B., Khamari L., Bhate J. (2021). Artificial Intelligence-Led Content Publishing, Metadata Creation, and Knowledge Discovery: In Quest of Sustainable and Profitable Business Models. Transforming Scholarly Publishing with Blockchain Technologies and AI.

[44] Shrivas M.K., Yeboah D. (2017). A Critical Review of Cryptocurrency Systems. Texila Int. J. Acad. Res..

[45] Parekh R., Patel N.P., Thakkar N., Gupta R., Tanwar S., Sharma G., Davidson I.E., Sharma R. (2022). DL-GuesS: Deep Learning and Sentiment Analysis-based Cryptocurrency Price Prediction. IEEE Access.

[46] Lo S.K., Xu X., Staples M., Yao L. (2020). Reliability analysis for blockchain oracles. Comput. Electr. Eng..

[47] Jain S., Felten E., Goldfeder S. (2018). Determining an optimal threshold on the online reserves of a bitcoin exchange. J. Cybersecur..

[48] Zhang B., Li X., Ren H., Gu J. (2019). Semantic Knowledge Sharing Mechanism Based on Blockchain. The International Conference on Natural Computation, Fuzzy Systems and Knowledge Discovery.

[49] Cai C., Zheng Y., Zhou A., Wang C. (2019). Building a secure knowledge marketplace over crowdsensed data streams. IEEE Trans. Dependable Secur. Comput..

[50] Singh S., Singh N. Blockchain: Future of financial and cyber security. Proceedings of the 2016 2nd International Conference on Contemporary Computing and Informatics (IC3I).

[51] Peng P., Tian Y., Xiang T., Wang Y., Pontil M., Huang T. (2017). Joint semantic and latent attribute modelling for cross-class transfer learning. IEEE Trans. Pattern Anal. Mach. Intell..

[52] Lv C., Xing Y., Lu C., Liu Y., Guo H., Gao H., Cao D. (2018). Hybrid-learning-based classification and quantitative inference of driver braking intensity of an electrified vehicle. IEEE Trans. Veh. Technol..

[53] Schluse M., Priggemeyer M., Atorf L., Rossmann J. (2018). Experimentable digital twins—Streamlining simulation-based systems engineering for industry 4.0. IEEE Trans. Ind. Inform..

[54] Qi Y., Xiao J. (2018). Fintech: AI powers financial services to improve people’s lives. Commun. ACM.

[55] Feurer M., Eggensperger K., Falkner S., Lindauer M., Hutter F. Practical automated machine learning for the automl challenge 2018. Proceedings of the International Workshop on Automatic Machine Learning at ICML.

